# Supplementary material for: Endogenous Bornavirus-like Elements in Bats: Evolutionary Insights from the Conserved Riboviral L-Gene in Microbats and Its Antisense Transcription in Myotis daubentonii
Source: Viruses. 2024 Jul 27;16(8):1210. doi: 10.3390/v16081210 (PMC11360350; doi:10.3390/v16081210)
Supplement: Supplementary file 1 [file viruses-16-01210-s001.zip › viruses-3012330-supplementary.pdf]

Supplement for Ritsch et al., Bornavirus-like elements in bats: Evolutionary insights from the conserved riboviral L-gene in microbats and its antisense transcription in Myotis daubentonii, Viruses 2024

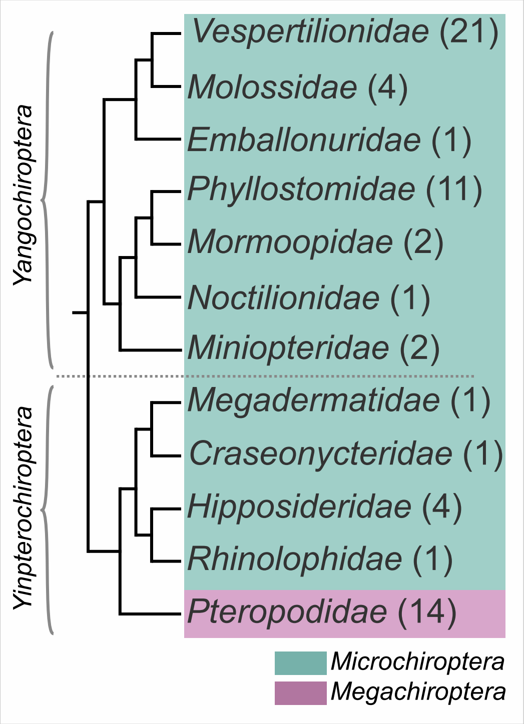

Figure S1. The phylogeny of bats is depicted, delineating the two suborders *Yangochiroptera* and *Yinpterochiroptera*. Historically, bats have been classified into *Microchiroptera* (microbats) and *Megachiroptera* (megabats), also represented here. The tree is resolved up to the family level and is adapted from the study by Agnarsson *et al.* [63]. In parentheses is the number of genomes used for this studies; also refer to Table 1

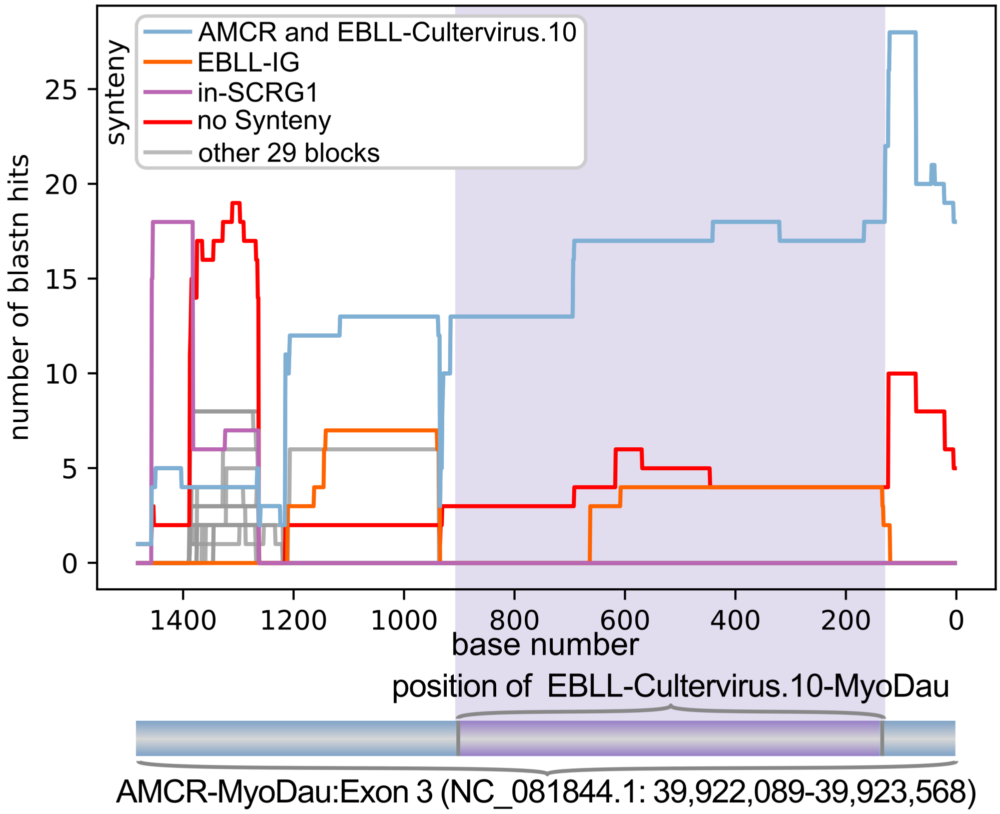

Figure S2. All blastn hits from Exon 3 in the various bat species were mapped back to the original Exon 3 sequence of *Myotis daubentonii* (the reference sequence). The hits were then grouped according to their synteny units; see Figure 4 and S3. Notably, in the region of the L protein (highlighted in purple), there are only three synteny blocks. The blue blocks represent hits from the newly described EBLL-Cultervirus.10-MyoDau (short: CV.10-MyoDau) in this paper, the orange blocks are from the previously described EBLL-IG. The red lines indicate hits for which no synteny assignment could be determined, often due to contigs being too short.

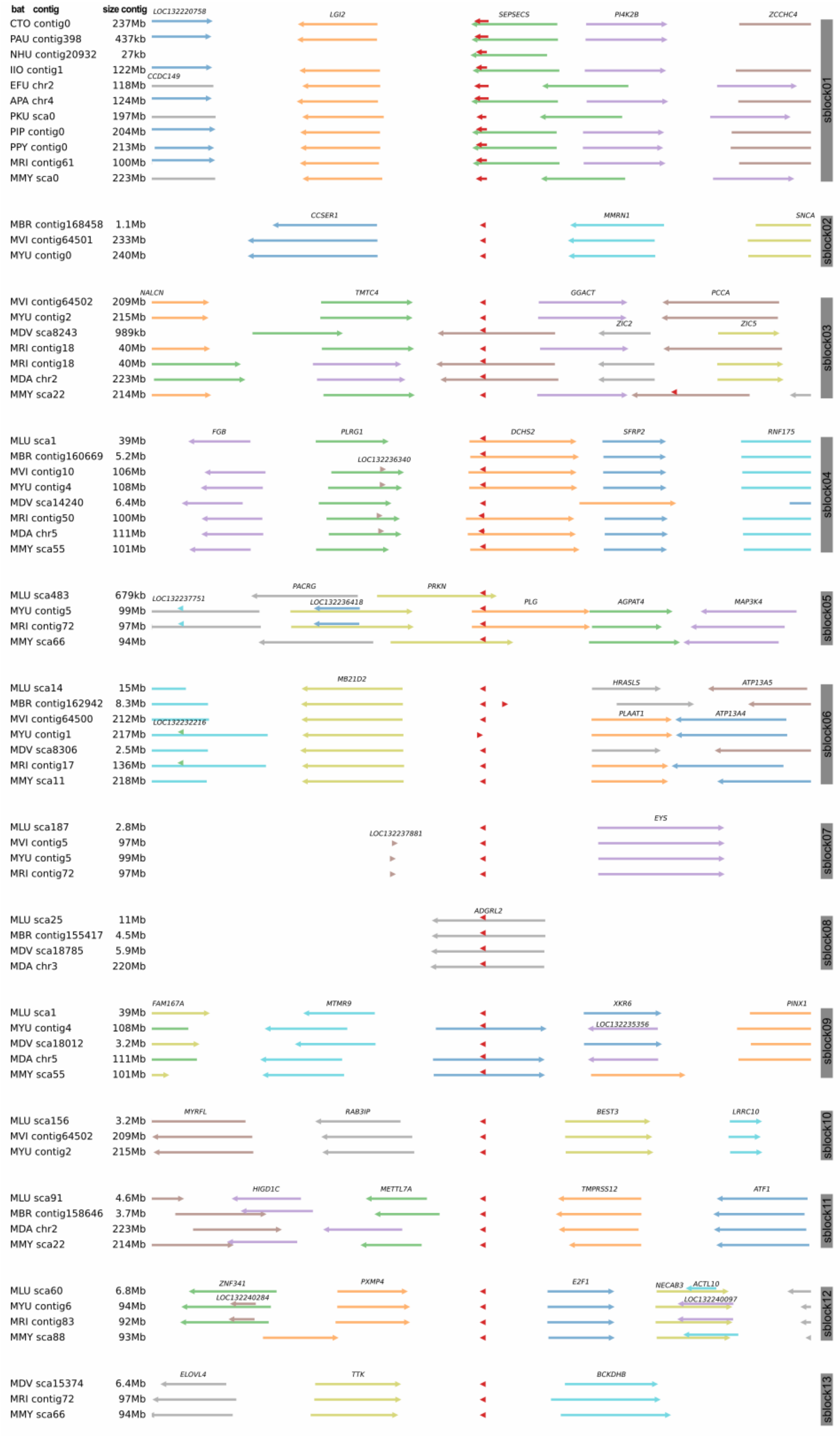

The figure will continue on the next page

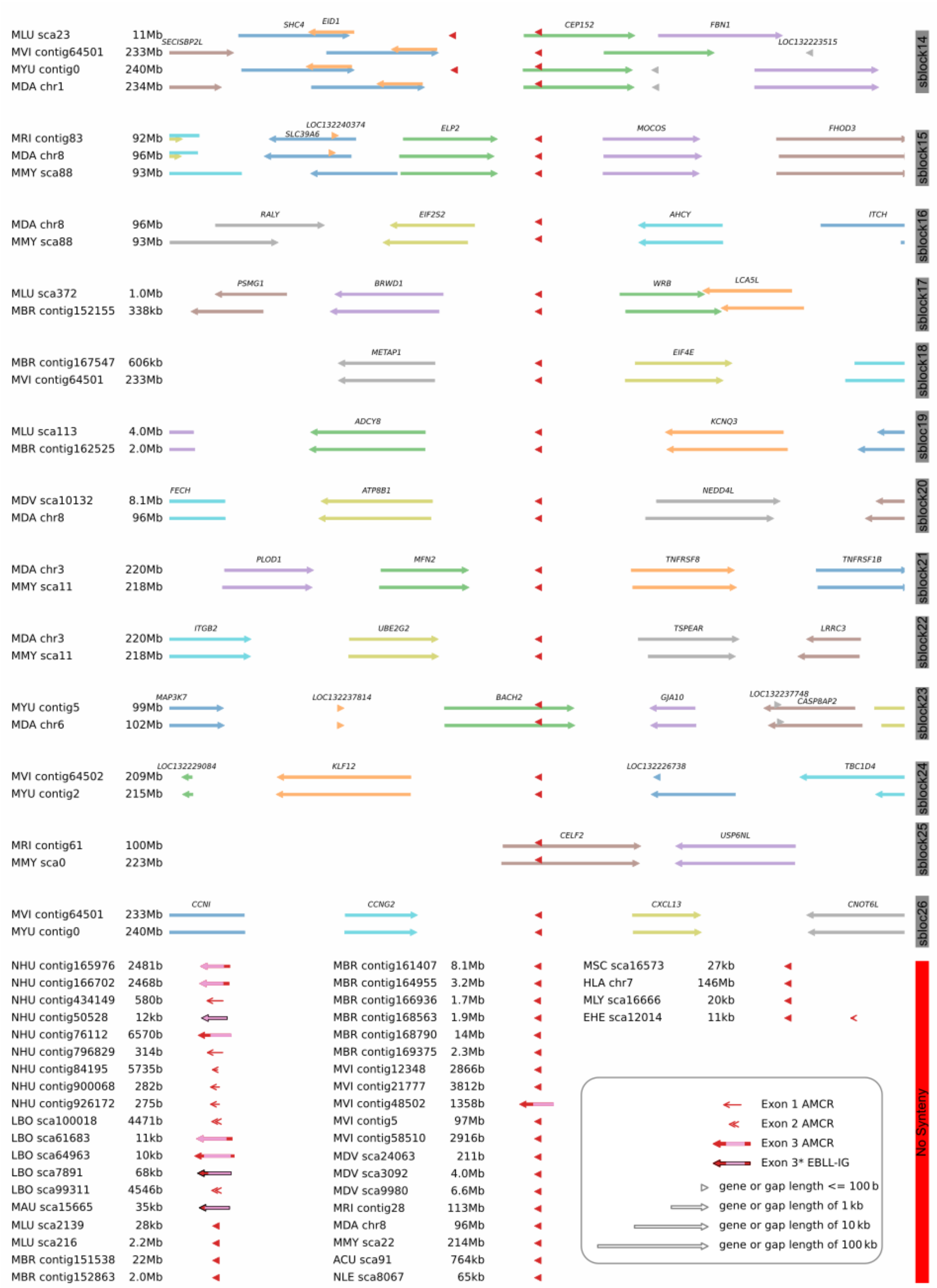

Figure S3. Here, the missing 26 synteny blocks are depicted, which were only hinted at in Figure 4, along with all the hits for which no synteny was found. The bat species, the contig's name, and the respective contig's size are provided for each hit. For a detailed description, see Figure 4.
